# Timed physical exercise does not influence circadian rhythms and glucose tolerance in rotating night shift workers: The EuRhythDia study

**DOI:** 10.1177/1479164120950616

**Published:** 2020-09-25

**Authors:** Juliane Hannemann, Anika Laing, Karin Glismann, Debra J Skene, Benita Middleton, Bart Staels, Nikolaus Marx, Peter J Grant, Massimo Federici, Josef Niebauer, Rainer Böger

**Affiliations:** 1 Institute of Clinical Pharmacology and Toxicology, University Medical Center Hamburg-Eppendorf, Hamburg, Germany; 2 Chronobiology, Faculty of Health and Medical Sciences, University of Surrey, Guildford, UK; 3 Univ. Lille, Inserm, CHU Lille, Institut Pasteur de Lille, U1011–EGID, Lille, France; 4 Department of Cardiology, University Medical Center Aachen, Aachen, Germany; 5 Leeds Institute of Cardiovascular and Metabolic Medicine, University of Leeds, Leeds, UK; 6 Center for Atherosclerosis, School of Medicine, University of Rome ‘Tor Vergata’, Rome, Italy; 7 University Institute for Preventive and Rehabilitative Sports Medicine, Paracelsus Medical University, Salzburg, Austria

**Keywords:** Night shift workers, glucose tolerance, diabetes mellitus, vascular stiffness, insulin, prospective study

## Abstract

**Objectives:**

Night shift workers are at cardiometabolic risk due to circadian misalignment. We investigated whether infrequent exercise before each night shift that intentionally would not improve physical performance improves glucose tolerance and 24-h blood pressure profiles and synchronizes circadian rhythms of melatonin and cortisol in rotating night shift workers.

**Methods:**

A total of 24 rotating night shift workers (mean age, 35.7 ± 11.8 years) were randomized to exercise or no intervention. Workers in the exercise group performed 15.2 ± 4.5 exercise sessions within 2 h before each night shift. Before and after 12 weeks of exercise intervention and 12 weeks after the intervention, spiroergometry, oral glucose tolerance testing and 24-h blood pressure profiles were performed. Plasma melatonin and cortisol levels were measured in 3-hourly intervals during one 24-h period on each study day.

**Results:**

Exercise did not significantly change serum glucose nor insulin levels during oral glucose tolerance testing. Timed physical exercise had no effect on physical performance, nor did it change the circadian rhythms of melatonin and cortisol or influence 24-h blood pressure profiles.

**Conclusion:**

Physical exercise before each night shift at a low intensity level that does not improve physical performance does not affect circadian timing, glucose tolerance or 24-h blood pressure profiles in rotating night shift workers.

## Introduction

The rates of obesity and type 2 diabetes mellitus (T2DM) are increasing^1^ [International Diabetes Federation (IDF) Diabetes Atlas 2017]. The associated morbidity and mortality are mainly driven by the resulting cardiometabolic diseases.^2^ Epidemiological evidence indicates that obesity-related insulin resistance predates the development of clinically overt T2DM by up to 20 years.^3^ As individuals progress from euglycemic insulin resistance to beta cell failure and clinically overt hyperglycemia, cardiovascular risk further increases, and the risk of microvascular complications develops.

In murine studies, genetic disruptions in circadian clock genes lead to the development of a phenotype similar to human type 2 diabetes/obesity.^4^ Although there are no studies in human subjects that causally relate clock genes to the development of T2DM, epidemiological data have shown that shift workers – who are known to experience disrupted circadian rhythms^5^ – are at increased risk of developing cardiometabolic disease.^6,7^ Disruption of circadian rhythms in night shift workers is thought to result from misalignment between the central clock that tunes metabolism to sleep mode in the evening hours, and external stimuli associated with the requirements of work that force the night shift worker to remain in active.^8^ Light, physical activity and stress hormones affect glucose metabolism directly and through the circadian clock in this group of workers. A prospective study that included 402 night shift workers and 336 daytime workers who were followed for 4 years uncovered a fivefold elevated risk of developing T2DM/obesity in night shift workers.^9^ A meta-analysis of nine cross-sectional studies including almost 14,000 participants and four prospective studies with almost 9500 participants found a 57% higher incidence of the metabolic syndrome in workers exposed to night shift work as compared to workers who had never been exposed to night shift work.^10^

Exercise has long been known to exert beneficial effects on cardiometabolic health,^11,12^ an effect that has mainly been linked to the endurance training effect of this intervention. However, physical exercise also plays an important role as a non-photic ‘zeitgeber’ for the internal clock.^13^ The EuRhythDia consortium set out to investigate whether modulation of the circadian clock through lifestyle intervention influenced circadian rhythms in night shift workers and, by this mechanism, improved insulin sensitivity in this risk group. We designed the present study in order to investigate whether timed physical exercise (immediately before each night shift) might act as a zeitgeber stimulus, reset the circadian clock, activate the circulatory and metabolic systems, and thereby ameliorate the disruptions that night shift work exerts on the synchronization of circadian rhythms, glucose regulation and blood pressure regulation. To avoid the well-described effect of improved physical performance on glucose control and blood pressure regulation, exercise was intentionally performed at a low frequency and was timed immediately before each night shift, in order to act as an impulse on the circadian timing system.^13^

In this study, we therefore investigated the effects of timed exercise intervention within a short, defined time frame of 2 h before each night shift over a period of 12 weeks on glucose metabolism, circadian rhythms and 24-h blood pressure in apparently healthy rotating night shift workers. During further 12 weeks without exercise intervention (wash-out), we aimed to study the reversibility of any changes caused by the exercise impulse.

## Methods and study design

### Study participants

Twenty-four apparently healthy individuals undergoing rotating night shift work were recruited into this study. Night shift workers were included if they had undergone rotating night shift work with at least three night shifts per month for a duration of at least 6 months. They were excluded if they had any severe somatic or psychiatric disease or signs of obstructive sleep apnoea, if they had been on a long-distance flight (three time zones or more) within 4 weeks before the start of the study or if they had taken melatonin supplements within 4 weeks before commencing the study. Caffeine intake above 750 mg/day and blood donation within 60 days before inclusion in the study were also exclusion criteria. All study participants gave their written informed consent to participate in the study. The study protocol was approved by the Ethics Committee of the Chamber of Physicians of Hamburg (decision no. PV4287).

### Study design

All study participants underwent an initial study visit comprising medical history, full clinical examination, blood sampling for routine laboratory tests, spiroergometry testing and an oral glucose tolerance testing (OGTT). The participants received a 24-h blood pressure measurement. Starting in the next morning at 08:00 am, all study participants were confined to an in-patient ward for a total of 26 h, during which blood samples were drawn in 3-h intervals for the determination of plasma melatonin and cortisol concentrations. Night shift workers were randomized to exercise or no-intervention groups, respectively, at the end of the first study day, and were instructed about study procedures and the details of the exercise programme (see below).

During 12 weeks, night shift workers allocated to the exercise group participated in supervised exercise sessions before each night shift, within a time frame of 2 h before the beginning of the respective night shift. Individuals allocated to the no-intervention group received no specific instructions and performed no specific physical exercise. All study participants were asked to maintain diaries regarding their daily activities, night shift schedules, sleeping behaviour and general well-being; however, food intake and its timing were not restricted.

After 12 weeks, post-treatment assessments following the same procedures as during the baseline assessment were performed. This study visit was scheduled within 48 h after the end of a night shift. Thereafter, study participants were asked to return after a further 12 weeks, during which no specific intervention was performed in any of the groups (wash-out phase). After a total of 24 weeks, within 48 h after the end of a night shift, a final assessment (wash-out examination) was performed in an identical manner as during the baseline and post-treatment assessments.

The primary outcome parameter was the change in the area under the serum glucose/time curve during OGTT before and after 12 weeks of exercise or no intervention. Secondary outcome parameters were the change in insulin during OGTT, the change in the homeostasis model assessment of insulin resistance (HOMA-IR) and quantitative insulin sensitivity check index (QUICKI) indices and the change in 24-h profiles of blood pressure and of melatonin and cortisol. Due to the intense phenotyping approach of our study, the number of study participants was limited, thereby limiting the power of our study to detect minor changes in the primary outcome parameter. Our study was powered to significantly detect a 56% change in the primary outcome parameter.

### Physiological examinations

All study participants underwent an OGTT during each clinical examination. After baseline blood sampling, 75 g of glucose was administered orally in 100 mL of water, and repeated blood sampling was performed at 30, 60, 90 and 120 min after glucose ingestion. Blood samples were centrifuged and serum was stored for glucose and insulin determination as described below.

Spiroergometry was performed at each clinical examination using a standard clinical spiroergometer (MetaSoft, Cortex, Leipzig, Germany). Starting load and increments were between 20 and 50 Watt (W) based on sex, age and training status to reach maximal exhaustion within 12–15 min. The same individual exercise protocol was used for all the subsequent assessments.^14^ Maximal exercise load was determined by maximal relative physical performance (W_max_), maximal oxygen uptake (VO_2max_), maximal heart rate (HR_max_) and maximal blood lactate concentration (LAC_max_).

Twenty-four hour blood pressure measurements were performed with the BPLab^®^ ambulatory blood pressure monitoring system (OOO Petr Telegin, Nizhny Novgorod, Russia), which includes analysis of 24-h profiles of arterial stiffness. For arterial stiffness determination, central systolic blood pressure (cSBP), central pulse pressure (cPP), augmentation index corrected to a normative heart rate of 75 beats/min (AIx75) and pulse wave velocity (PWV) were analysed. Starting in the morning of a day that was followed by a night shift, study participants received actigraphy recorders for 72 h (MotionWatch 8, CamNtech Ltd, Cambridgeshire, United Kingdom) during the week before each clinical investigation.

### Exercise protocol

It was the aim of the exercise intervention to not induce relevant adaptations of physical performance, in order to be able to discriminate the potential effects of exercise on glucose and blood pressure regulation via its influence on circadian timing from the well-known improvements in cardiometabolic status induced by improved physical fitness. Therefore, the exercise protocol was intentionally designed at a frequency and intensity level at which improvement in physical performance was not expected; it was a high-intensity interval training (HIT) programme based on the results of the first spiroergometry test^15^ consisting of four high-intensity intervals at 85%–95% of maximal heart rate (4 min each), separated by recovery phases at 65%–75% of HR_max_ (3 min each). A warm-up and a cool-down phase (5 min each at 60%–65% of HR_max_) framed the 35-min exercise session. This supervised exercise was to be performed within 2 h before the beginning of each night shift.

### Biochemical analyses

Serum glucose and glycated haemoglobin (HbA_1c_) concentrations as well as routine laboratory values were measured by standard clinical chemistry methods in the local laboratory. Blood lactate was determined with a Biosen lactate analyzer (EKF Diagnostics, London, United Kingdom). Serum insulin was measured by enzyme-linked immunoassay (Mercodia, Uppsala, Sweden). Plasma melatonin and cortisol concentrations were measured in 3-h lithium heparin plasma samples by specific radioimmunoassays with reagents obtained from Stockgrand Ltd. (University of Surrey) as described previously (Gunn et al. 2016).^16^ All samples from one participant were run in the same assay and measured in duplicate. Limit of detection of the melatonin and cortisol assay (mean ± SD) was 2.8 ± 1.0 pg/mL and 1.6 ± 0.7 nmol/L, respectively. For both assays, the interassay coefficients of variation (CVs) of low, medium, high and very high QCs were <13% (range: 4.9%–12.7%; N = 30 and 34, respectively).

### Calculation of insulin resistance indices

The HOMA-IR is a widely used parameter to estimate the extent of insulin resistance in an individual, based on fasting insulin and fasting glucose concentrations, according to the formula HOMA-IR = fasting insulin (mU/L)*fasting glucose (mg/dL)/405.^17,18^ The HOMA-IR shows close correlation with the results of with euglycemic clamp, and it is suitable to detect early insulin resistance in individuals with normal fasting glucose levels.^19^

Like HOMA-IR, QUICKI uses fasting glucose and insulin concentrations^20^ in the formula QUICKI = 1/[log(fasting insulin) + log(fasting glucose)]. QUICKI results in a more reliable estimation of insulin resistance in individuals with or without obesity and in patients with clinically overt diabetes mellitus.^20^

### Statistical analyses

All variables were tested for normal distribution using the Kolmogorov–Smirnov test. Differences between groups were tested for significance using either the non-parametric Mann–Whitney U test for two groups or two-tailed t-test for normally distributed variables. Repeated measurement analysis of variance (ANOVA) with Bonferroni correction was used for time courses of parameters measured at baseline, at 12 and at 24 weeks. Data are presented as median with 25th and 75th percentiles, or as mean with standard error of the mean. For all tests, *p* < 0.05 was considered significant.

## Results

### Baseline characteristics of the study groups

The baseline demographic and anthropometric characteristics of the study participants are summarized in Table 1. The night shift workers had a median duration of work including night shifts of 8.0 years [interquartile range (IQR), 4.3–21.0]; their median number of night shifts per month during their current occupation was 6.0 (IQR, 5.0–7.0). All subjects were normotensive (Table 1).

**Table 1.** Demographic and anthropometric characteristics of the study participants.

	Night shift workers		
Demographics	Exercise	No exercise	*p*
N	12	12	ns
Male sex, n (%)	5 (42)	3 (25)	ns
Age (years)	34.2 ± 8.6	37.3 ± 13.7	ns
Anthropometrics			
BMI (kg/m^2^)	26.8 ± 5.6	27.8 ± 6.9	ns
Waist/Hip ratio	0.9 ± 0.1	0.9 ± 0.1	ns
Work history			
No. of years on night shift [median (IQR)]	9.5 (4.5; 20.5)	6.0 (4.3; 21.0)	ns
No. of night shifts/month [median (IQR)]	6.0 (5.0; 7.0)	6.0 (5.0; 8.5)	ns
Health care provider (nurse, physician), n (%)	7 (58)	7 (58)	ns
Firefighter, police officer, n (%)	3 (25)	2 (17)	ns
Worker in industry, employee, n (%)	1 (8.5)	3 (25)	ns
Bus or taxi driver, n (%)	1 (8.5)	0 (0)	ns
Cardiometabolic parameters			
Systolic blood pressure (mm Hg)	122.5 ± 9.8	120.7 ± 7.3	ns
Diastolic blood pressure (mm Hg)	74.4 ± 8.4	76.8 ± 6.7	ns
Heart rate (1/min)	66.2 ± 13.3	71.5 ± 6.2	ns
Fasting glucose (mg/dL)	94.4 ± 23.9	86.4 ± 8.3	ns
HbA_1c_ (%)	5.2 ± 0.6	4.9 ± 0.3	ns
Physical training status			
VO_2max_ (mL/min/kg)	35.8 ± 7.3	30.1 ± 6.8	ns
Relative physical performance (W/kg)	2.7 ± 0.7	2.2 ± 0.6	ns
Maximal heart rate (beats/min)	172.6 ± 11.0	172.8 ± 17.5	ns

BMI: body mass index; IQR: interquartile range; HbA_1c_: glycated haemoglobin; VO_2max_: maximal oxygen uptake.

Data are given as mean ± standard deviation, unless indicated otherwise.

Although there was a slight trend towards better training status in individuals randomized to the exercise group than to the control group, both groups did not differ significantly: baseline peak VO_2_ was 35.8 ± 3.7 mL/min/kg in the exercise group versus 29.9 ± 7.1 mL/min/kg in the no-intervention group (*p* = ns). Similarly, relative physical performance was 2.7 ± 0.7 versus 2.2 ± 0.6 W/kg, respectively (*p* = ns), and maximal heart rate was 172.6 ± 11.0 versus 172.3 ± 17.5 beats/min (*p* = ns).

### Effects of timed physical exercise on exercise performance

Study participants worked an average of 18.1 ± 4.0 night shifts during the 12-weeks study period. Adherence of the subjects to the exercise protocol was 86.4% ± 9.3%, with an average of 15.2 ± 4.5 exercise sessions performed during the 12-weeks period. Twelve weeks of exercise intervention before each night shift did not significantly increase peak VO_2_, maximal workload, maximal heart rate or lactate threshold during exercise, nor was there any change in exercise parameters after 12 weeks of wash-out (Table 2).

**Table 2.** Physical performance of the study participants during ergospirometry.

	Baseline	12 weeks	24 weeks
VO_2max_ (mL/min/kg)			
Exercise	35.8 ± 7.7	34.5 ± 9.6	33.5 ± 9.2
No intervention	30.1 ± 7.1	29.3 ± 7.3	28.6 ± 7.8
Maximal workload (W/kg)			
Exercise	2.7 ± 0.7	2.8 ± 0.8	2.7 ± 0.7
No intervention	2.2 ± 0.6	2.2 ± 0.6	2.2 ± 0.6
HR_max_ (1/min)			
Exercise	172.6 ± 11.5	170.5 ± 11.7	172.9 ± 9.5
No intervention	172.8 ± 18.3	171.4 ± 14.0	172.1 ± 17.4
Lactate threshold (W/kg)			
Exercise	2.3 ± 0.6	2.4 ± 0.8	2.3 ± 0.7
No intervention	1.8 ± 0.4	1.8 ± 0.4	1.8 ± 0.4

VO_2max_: maximal oxygen uptake; HR_max_: maximal heart rate.

Data are given as mean ± SD. There were no statistically significant differences between groups or within groups.

Actigraphy recordings showed that night shift workers had quite irregular sleep patterns with lots of nighttime physical activity on off-night shift days during the baseline assessment period. After 12 weeks of exercise intervention, physical activity during nights off-work was much lower; however, 12 weeks after the end of the intervention, the nightly activity profile had returned to baseline status. The means of 72-h actigraphy recordings are shown in Supplemental Figure 1(a) to (c) for the exercise group and in Supplemental Figure 1(d) to (f) for the no-intervention group.

### Effects of timed physical exercise on glucose tolerance

**Figure 1.**
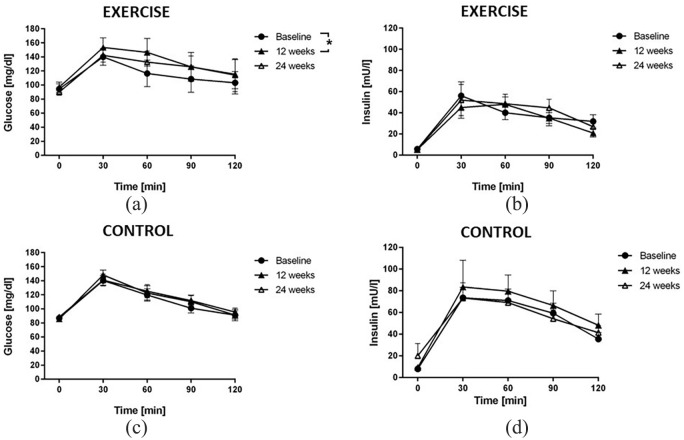
Plasma glucose and insulin concentrations during oral glucose tolerance testing (OGTT). Lines represent the time courses of mean glucose concentration (a and b) and mean insulin concentration (c and d) in rotating night shift workers undergoing exercise intervention (a and c) or no intervention (control; b and d) before the night shift. OGTTs were performed at baseline, after 12 weeks of controlled intervention and after 12 more weeks of wash-out. Blood samples were drawn at baseline (08:00 h) and at 30, 60, 90 and 120 min after the oral ingestion of 75 g of glucose in 100 mL of tap water. Data are mean ± SEM of 12 participants.

The time courses of serum glucose and insulin concentrations during the OGTT were not significantly different between both groups of night shift workers at baseline. There was a significant increase in the area under the glucose concentration curve during the OGTT after 12 weeks of exercise intervention [Figure 1(a)], while insulin levels did not significantly change [Figure 1(c)]. No significant differences were observed for glucose and insulin in the control group [Figure 1(b) and (d)].

Exercise intervention had no significant effect on HOMA-IR, QUICKI index or HbA_1c_ (Table 3).

**Table 3.** Indices and biochemical markers of glucose control.

	Baseline	12 weeks	24 weeks
HOMA-IR			
Exercise	1.3 ± 0.6	1.2 ± 0.4	1.2 ± 0.6
No intervention	1.7 ± 1.0	2.1 ± 1.2	1.9 ± 1.0
QUICKI index			
Exercise	0.37 ± 0.03	0.38 ± 0.02	0.38 ± 0.03
No intervention	0.36 ± 0.04	0.36 ± 0.03	0.36 ± 0.03
HbA_1c_ (%)			
Exercise	5.2 ± 0.6	5.2 ± 0.7	5.2 ± 0.6
No intervention	4.9 ± 0.2	5.1 ± 0.3	5.0 ± 0.2

HOMA-IR: homeostasis model assessment of insulin resistance; QUICKI: quantitative insulin sensitivity check index; HbA_1c_: glycosylated haemoglobin.

Data are given as mean ± SD. There were no statistically significant differences between groups or within groups.

### Effects of timed physical exercise on diurnal rhythms of melatonin and cortisol

**Figure 2.**
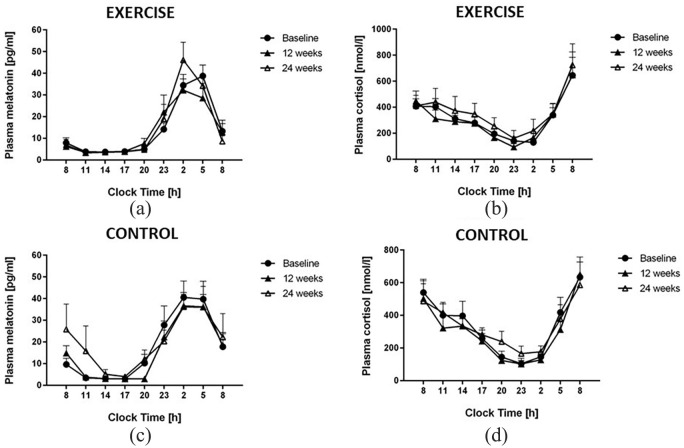
Time profiles of plasma melatonin in rotating night shift workers allocated to the exercise intervention (a) and no-intervention groups (b), and of plasma cortisol in night shift workers allocated to the exercise intervention (c) and no-intervention groups (d). There were no significant differences between both intervention groups nor any time-dependent differences within each of the groups. Data are mean ± SEM of 12 participants.

There was no significant difference between the baseline circadian profiles of melatonin and cortisol in the exercise and no-intervention groups. Exercise intervention did not cause any significant change in mean melatonin concentration or in the timing of the circadian rhythm of melatonin or cortisol plasma concentrations [Figure 2(a) to (d)].

### Effects of timed physical exercise on 24-h profiles of blood pressure and arterial stiffness

**Figure 3.**
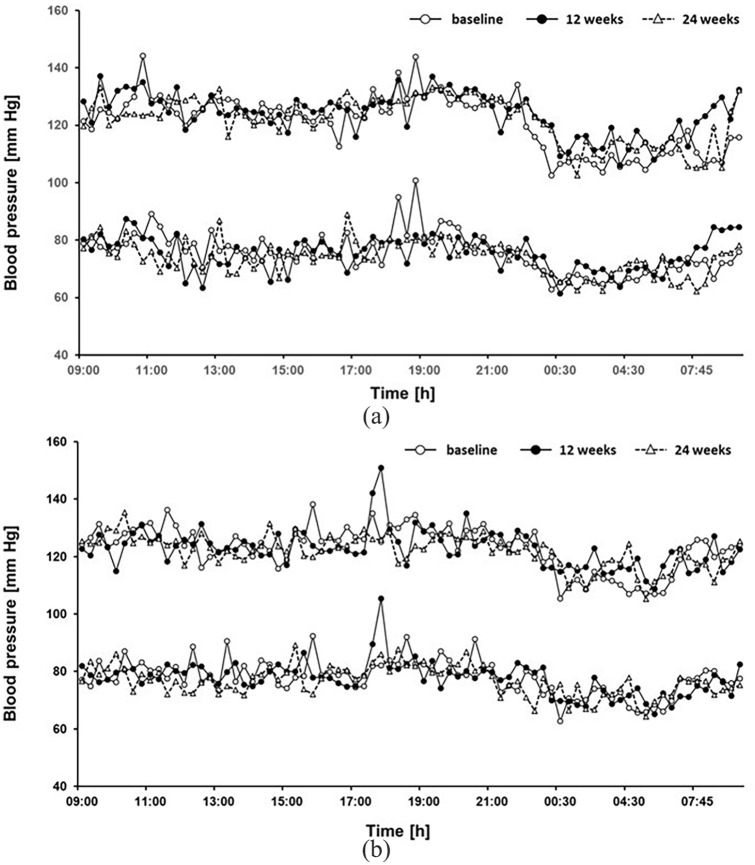
Twenty-four hour blood pressure profiles in rotating night shift workers allocated to the exercise intervention (a) and no-intervention groups (b). Data are mean of n = 12 participants. Variability indices are not plotted to enhance readability of the graph.

Twenty-four hour blood pressure profiles were not significantly different between the exercise and no-intervention groups. Twelve weeks of timed exercise intervention did not significantly affect 24-h blood pressure profile [Figure 3(a) and (b)]. Moreover, PWV, central aortic blood pressure and augmentation index were not significantly different between the groups nor affected by the exercise intervention (Table 4).

**Table 4.** Parameters of daytime and nighttime arterial stiffness.

	Baseline	12 weeks	24 weeks
Daytime systolic blood pressure (mm Hg)			
Exercise	123.7 ± 9.1	127.1 ± 11.5	125.8 ± 10.4
No intervention	126.2 ± 12.9	123.8 ± 9.5	123.7 ± 4.8
Nighttime systolic blood pressure (mm Hg)			
Exercise	111.3 ± 6.9	117.2 ± 7.2	117.6 ± 11.8
No intervention	114.3 ± 10.0	118.1 ± 12.1	117.3 ± 11.5
Daytime diastolic blood pressure (mm Hg)			
Exercise	76.4 ± 6.7	76.7 ± 6.8	75.0 ± 4.6
No intervention	79.9 ± 6.3	78.4 ± 4.8	78.6 ± 6.7
Nighttime diastolic blood pressure (mm Hg)			
Exercise	68.6 ± 8.5	70.2 ± 6.6	69.9 ± 5.6
No intervention	71.3 ± 7.3	73.2 ± 8.0	72.3 ± 10.0
Daytime cSBP (mm Hg)			
Exercise	112.6 ± 7.6	115.6 ± 10.5	114.6 ± 8.8
No intervention	115.5 ± 11.4	113.3 ± 7.6	113.2 ± 4.8
Nighttime cSBP (mm Hg)			
Exercise	102.5 ± 7.3	107.9 ± 6.6	108.4 ± 11.3
No intervention	105.7 ± 9.1	108.3 ± 10.9	108.2 ± 11.4
Daytime cPP (mm Hg)			
Exercise	34.3 ± 5.0	37.3 ± 8.1	38.1 ± 7.8
No intervention	33.6 ± 7.0	32.9 ± 5.8	33.0 ± 4.9
Nighttime cPP (mm Hg)			
Exercise	32.6 ± 4.5	36.3 ± 5.8	37.2 ± 7.4
No intervention	32.9 ± 6.4	33.4 ± 5.1	34.5 ± 5.0
Daytime Aix			
Exercise	−45.7 ± 15.7	−47.3 ± 18.4	−49.8 ± 17.3
No intervention	−47.0 ± 14.4	−45.6 ± 15.5	−48.2 ± 11.2
Nighttime Aix			
Exercise	−46.6 ± 11.5	−44.8 ± 20.6	−47.1 ± 15.3
No intervention	−40.5 ± 18.0	−43.3 ± 20.4	−42.7 ± 19.6
Daytime PWV (norm)			
Exercise	9.0 ± 1.5	9.1 ± 1.1	9.2 ± 1.4
No intervention	8.2 ± 1.5	8.6 ± 2.1	9.7 ± 1.6
Nighttime PWV (norm)			
Exercise	9.5 ± 1.2	9.5 ± 1.3	9.1 ± 1.5
No intervention	9.2 ± 1.5	9.3 ± 1.8	9.4 ± 1.8

cSBP: central aortic systolic blood pressure; cPP: central aortic pulse pressure; Aix: augmentation index; PWV (norm): pulse wave velocity normalized to systolic blood pressure of 100 mm Hg and heart rate of 60/min.

Data are given as mean ± SD. There were no statistically significant differences between groups or within groups.

## Discussion

The main finding of our study is that timed exercise sessions before each night shift at a low intensity level that does not improve physical exercise capacity has no significant effect on glucose tolerance, 24-h blood pressure profiles and the circadian rhythms of melatonin and cortisol in rotating night shift workers. Physical exercise improves cardiovascular function and glucose control in individuals at-risk of cardiovascular disease and type II diabetes. Both, regular continuous moderate exercise and high-intensity exercise positively influence cardiovascular function, blood pressure and insulin sensitivity in a variety of populations.^11,15,21^ These beneficial effects of physical exercise have been explained by increased calorie consumption, enhanced muscle performance, improved glucose utilization and improved endothelial function resulting in reduced peripheral insulin resistance. The extent of cardiometabolic benefit induced by exercise intervention has been related to the improvement in physical performance in most studies.

The environmental light–dark cycle is the key entrainment signal for circadian rhythms in human subjects, while other, non-photic stimuli are believed to play minor roles, including social activities, the timing of food ingestion and the more or less tight schedule of everyday activities.^22,23^ Beyond its training effects on physical performance, physical exercise exerts a non-photic entraining effect on the synchronization of circadian rhythms.^13^ However, evidence supporting this notion mainly originates from animal studies (reviewed by Redlin and Mrosovsky)^24^ and from studies with human subjects in closed experimental facilities under continuous dim light conditions, thereby excluding the circadian rhythm-entraining effect of daylight and other external stimuli.^22,25^

Our present study was specifically designed to take advantage of the supposed synchronizing effect of physical exercise to influence the phasing of circadian rhythms and thereby improve glucose control in night shift workers under real-life conditions. The intensity of the exercise was set to avoid an improvement in exercise capacity over time, in order to selectively study the influence of physical exercise as a zeitgeber for the timing of the circadian clock and – exclusively through this mechanism – for glucose metabolism. The timing of the exercise was set to a time window of 2 h before each night shift, assuming that this would phase-delay circadian rhythms and allow participants to stay alert for longer during the ensuing night shift.^22,25–29^ All exercise sessions were performed on the University Medical Center campus under supervision by study personnel; therefore, adherence to the exercise protocol could be calculated with high reliability as 86.4%, that is, 15.2 ± 4.5 exercise sessions during the 12-weeks intervention period.

The lack of effect of the exercise intervention on glucose metabolism, circadian rhythms and 24-h blood pressure control in our study population was corroborated by numerous different read-outs: first, the circadian timing of melatonin and cortisol – two hormones with well-described circadian rhythms directly driven by the circadian oscillator in the hypothalamic suprachiasmatic nuclei – remained unchanged after 12 weeks of exercise intervention. Second, the 24-h blood pressure profiles were virtually unchanged before and after the exercise intervention period. This was paralleled by a lack of effect of the exercise on parameters of arterial stiffness. Finally, glucose control was measured by OGTT with subsequent analysis of both, glucose and insulin, and showed an increase instead a decrease in serum glucose at the end of the exercise intervention, and no significant change in insulin levels throughout the study.

While as few as 4 days of simulated shift work were shown to reduce insulin sensitivity in healthy human subjects,^30^ a benchmark mean duration of as many as 25 years was calculated to be associated with an increase in HbA_1c_ by 30% or more in a 14-year cohort study of 7104 Japanese workers.^31^ Other investigators reported the presence of metabolic syndrome in permanent night shift workers.^32^ Therefore, one reason for the absence of effect of the exercise intervention chosen by us on cardiometabolic outcome parameters may lie in the characterization of the night shift workers included in our study. In agreement with many laboratory and field studies of shift workers, we did not observe any disruption of the circadian timing of melatonin and cortisol rhythms, nor did we observe major variability in these values, which might have suggested great variation within the study group. Moreover, the median number of night shifts per month was six, suggesting that circadian strain may have been too low with this ‘dose’ of night shifts to discern a significant disruption of circadian rhythms at baseline.

We also observed a physiological dipper effect of blood pressure in most of the study participants, suggestive of a general absence of circadian disruption of blood pressure regulation.^33^ In previous studies, the level of circadian misalignment in night shift workers was associated with the intensity of the night shift work. Specifically, Kitamura and co-workers reported that the circadian rhythm of blood pressure is transformed from a dipper pattern during phases of day work to a non-dipper pattern on the first day on a night shift pattern, but restored towards a dipper pattern after four nights of work,^34^ suggesting a complex relationship between the length of night shift work and circadian control of blood pressure. The workers included in our study performed a median of six night shifts per month and had been night shifts for a median of 8 (4–21) years. We therefore speculate that despite the long-term duration of rotating night shift work, about six solitary night shifts per month are too few to cause sustained disruption of circadian rhythms.

Our study has several strengths and limitations. First, the small number of patients limited our ability to detect minor changes in outcome parameters. This was counterbalanced by the intense phenotyping that we performed for one full 24-h cycle during each clinical investigation. Second, in designing the exercise intervention, we had to weigh the pros and cons of a high-intensity exercise intervention under best possible control of other stimuli known to entrain circadian rhythms against a lower intensity exercise intervention that would grant better acceptability in the community. We decided for an intervention scheme that would grant easy implementation in everyday worklife routines, even if this meant not to control for light intensities and food intake. The results of our study may thus not extend to all types and intensities of exercise intervention. Finally, we observed that the baseline level of physical fitness was slightly, although not significantly better in the workers allocated to the exercise intervention group than in the no exercise control group. This may have contributed to a lesser effect of the exercise programme within the study on the background of better trained individuals.

In a parallel study performed within the collaborative EuRhythDia project, Schäfer and co-workers performed exercise intervention in 64 night shift workers randomized to an exercise programme (n = 52) or no exercise control (n = 12). In that study, study participants in the exercise intervention group performed a mean 13.5 ± 4.0 exercise sessions during 12 weeks, that is, a similar number of exercise sessions like in the present study. A small but significant improvement in exercise capacity was observed after 12 weeks of intervention, and the exercise intervention caused a significant improvement in arterial stiffness.^35^ Both effects were reversible after subsequent 12 weeks of wash-out. The main differences between our present study and the study by Schäfer and co-workers is the smaller average number of night shifts undertaken during the exercise intervention period, which resulted in fewer exercise sessions and a lack of effect on physical exercise performance. This might explain the difference in outcome between both these studies and suggests that the potential benefits of high-intensity interval exercise on cardiometabolic disease risk depend on the ability of this intervention to improve physical exercise capacity.

Taken together, these studies clearly lead to the conclusion that physical exercise at a low intensity level as used in this study, which did not elicit improvements in exercise capacity, does not result in any effect on cardiometabolic control in rotating night shift workers with a relatively low night shift strain.

**Key messages**

- Moderate intensity rotating night shift work has only a mild effect on glucose tolerance and disruption of circadian rhythms of melatonin and cortisol.
- Timed physical exercise before each night shift at an intensity level that does not improve physical exercise capacity has no significant effect on glucose tolerance, 24-h blood pressure profiles and the circadian rhythms of melatonin and cortisol in rotating night shift workers.
- Timed physical exercise does not significantly affect arterial stiffness nor central aortic pressures.

## Supplemental Material

2020-07-06_-_DVDRes_-_Supplementary_Figures – Supplemental material for Timed physical exercise does not influence circadian rhythms and glucose tolerance in rotating night shift workers: The EuRhythDia study Click here for additional data file. Supplemental material, 2020-07-06_-_DVDRes_-_Supplementary_Figures for Timed physical exercise does not influence circadian rhythms and glucose tolerance in rotating night shift workers: The EuRhythDia study by Juliane Hannemann, Anika Laing, Karin Glismann, Debra J Skene, Benita Middleton, Bart Staels, Nikolaus Marx, Peter J Grant, Massimo Federici, Josef Niebauer and Rainer Böger in Diabetes & Vascular Disease Research
