# Supplementary material for: Timed physical exercise does not influence circadian rhythms and glucose tolerance in rotating night shift workers: The EuRhythDia study
Source: Diab Vasc Dis Res. 2020 Sep 25;17(5):1479164120950616. doi: 10.1177/1479164120950616 (PMC7919228; doi:10.1177/1479164120950616)
Supplement: 2020-07-06_-_DVDRes_-_Supplementary_Figures – Supplemental material for Timed physical exercise does not influence circadian rhythms and glucose tolerance in rotating night shift workers: The EuRhythDia study [file 2020-07-06_-_DVDRes_-_Supplementary_Figures.pdf]

Timed physical exercise does not influence circadian rhythms and glucose tolerance in rotating night shift workers – the EuRhythDia study.

Juliane Hannemann, Anika Laing, Karin Glismann, Debra J Skene, Benita Middleton, Bart Staels, Nikolaus Marx, Peter J Grant, Massimo Federici, Josef Niebauer, Rainer Böger

Supplementary Figure

72-hour actigraphy recordings of physical activity profiles.

Shift workers received actigraphy recorders for 72 hours, beginning in the morning before a night shift, at the baseline examination, after 12 weeks of exercise intervention, and 12 weeks after the end of exercise intervention (24 weeks). The graphs show mean activity profiles recorded from 12 rotating night shift workers per group.

a-c) Actigraphy recordings in the exercise intervention group;

d-f) actigraphy recordings in the no intervention group.

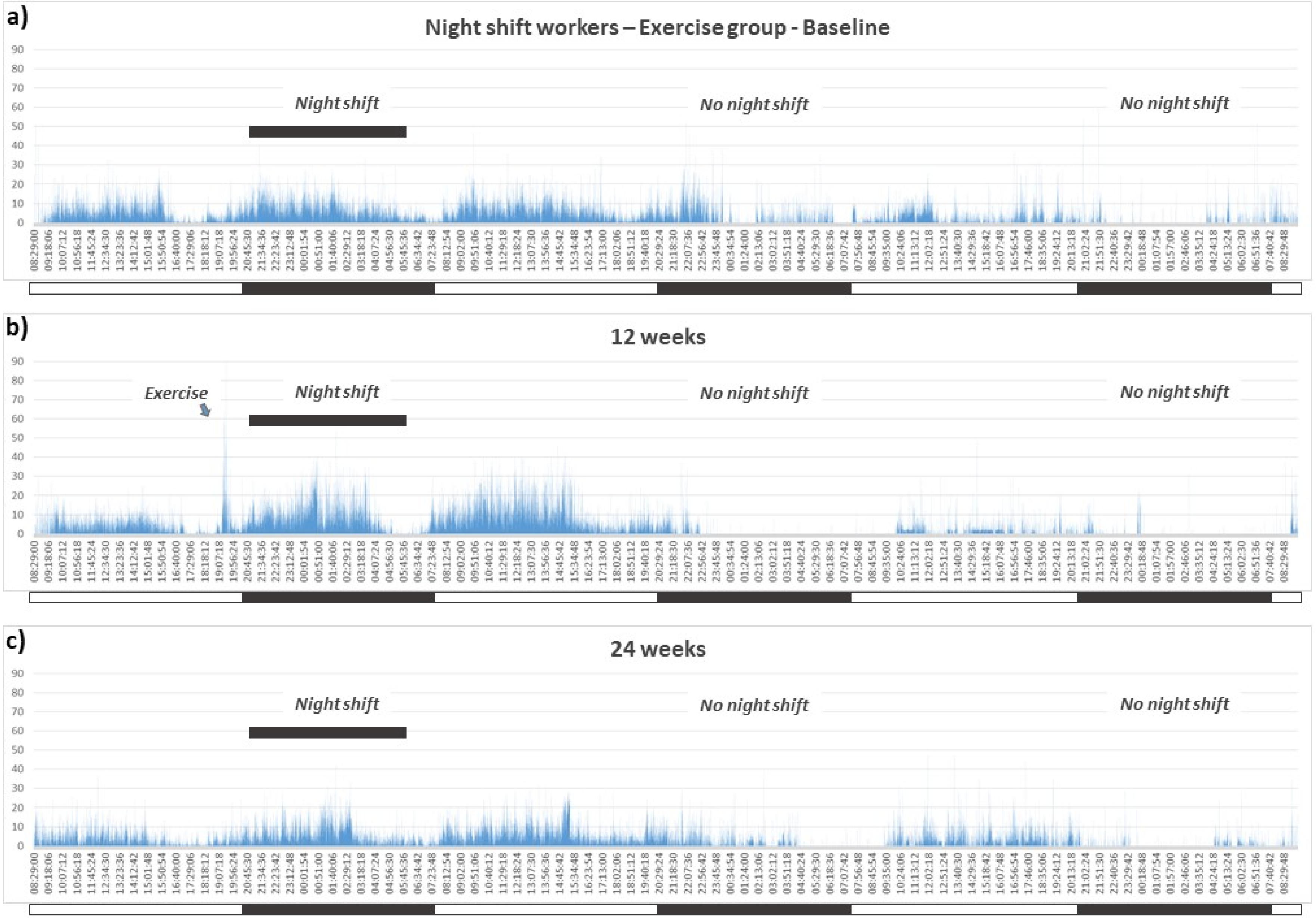

a)
Night shift workers – Exercise group - Baseline
Night shift
No night shift
No night shift
b)
12 weeks
Exercise
Night shift
No night shift
No night shift
c)
24 weeks
Night shift
No night shift
No night shift

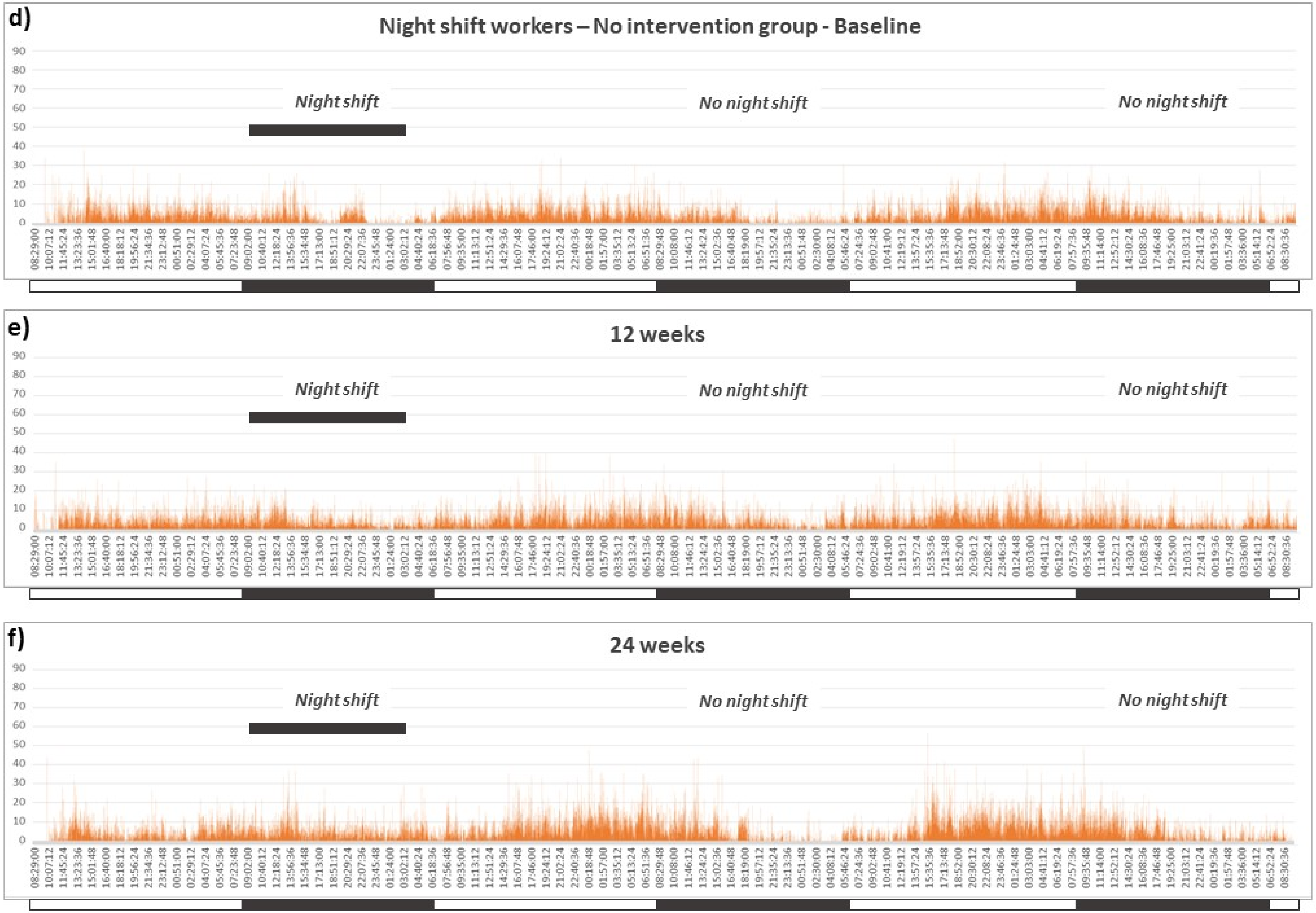
d)
Night shift workers – No intervention group - Baseline
Night shift
No night shift
No night shift
e)
12 weeks
Night shift
No night shift
No night shift
f)
24 weeks
Night shift
No night shift
No night shift
0
10
20
30
40
50
60
70
80
90
